# Retention of Vitamins A and D_3_ in Fortified Soybean Oil During Ethiopian Traditional Cooking and Shelf‐Life Stability During Storage

**DOI:** 10.1002/fsn3.70843

**Published:** 2025-09-03

**Authors:** Teklebrhan Gidey, Tilahun Bekele, Paulos Getachew

**Affiliations:** ^1^ Department of Food Science and Post‐Harvest Technology, College of Dry Land Agriculture and Natural Resources Mekelle University Mekele Ethiopia; ^2^ Center for Food Science and Nutrition, College of Natural and Computational Sciences Addis Ababa University Addis Ababa Ethiopia

**Keywords:** retention, shelf life, vitamin A, vitamin D_3_

## Abstract

Vitamin A and vitamin D_3_ deficiencies continue to pose serious health challenges, especially in low‐ and middle‐income countries like Ethiopia. One promising way to tackle this issue is by fortifying everyday foods—such as cooking oils—with essential nutrients to help improve the overall intake across communities. This study investigated the stbility of vitamins A and D_3_ in fortified soybean oil during common Ethiopian cooking practices and over time during storage. To test the vitamins' shelf life, the fortified oil was stored for 6 months at two temperatures: room temperature (25°C) and a warmer 37°C. As expected, both vitamins became less stable; the longer they were stored, and the higher the temperature, the greater the loss. Notably, vitamin D_3_ broke down much more than vitamin A under the same conditions. After 6 months, vitamin A levels dropped by just over 5% at room temperature and nearly 9% at 37°C. Meanwhile, vitamin D_3_ levels fell sharply by 59% and 62% at room temperature and 37°C, respectively. The study also examined how cooking affected vitamin levels. Food was prepared using the fortified oil at 250°C for 80 min. Even under these high‐heat conditions, more than 60% of vitamin A and a remarkable 97% of vitamin D_3_ were retained. These results show that vitamin A is more sensitive to heat, while vitamin D_3_ is more vulnerable to long‐term storage.

## Graphical abstract

Vitamin A and vitamin D_3_ deficiencies continue to pose serious health challenges in Ethiopia. Both vitamins became less stable the longer they were stored, and the higher the temperature, the greater the loss. Vitamin D_3_ broke down much more than vitamin A under the same conditions. Vitamin D_3_ levels fell sharply by 59% and 62% at room temperature and 37°C, respectively. Under high‐heat cooking, 60% of vitamin A and 97% of vitamin D_3_ were retained.

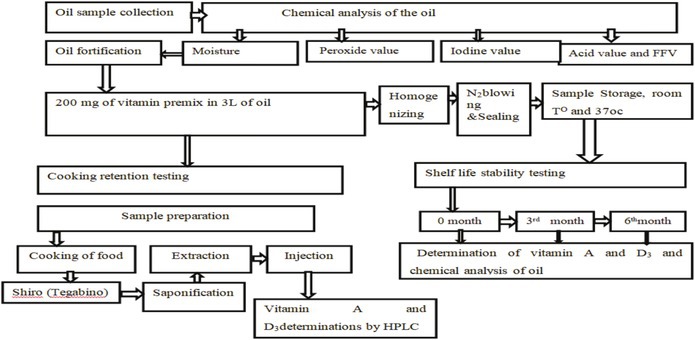

## 1 Introduction

Vitamin A and vitamin D_3_ deficiencies remain significant public health concerns, particularly in low‐ and middle‐income countries such as Ethiopia. Micronutrient malnutrition, often referred to as “hidden hunger,” affects millions around the world and contributes to a range of serious health problems, including weakened immune systems, night blindness, rickets, and increased mortality rates—especially among children and pregnant women (WHO 2022). In response to this ongoing challenge, food fortification has emerged as one of the most effective, sustainable, and affordable interventions. Fortifying widely consumed foods, such as edible oils, offers a promising strategy for improving micronutrient intake across entire populations (Das et al. 2019). The government of the Federal Democratic Republic of Ethiopia ratified that fortification of vegetable oil with vitamin A and D_3_, effective from 2011 E.C. to reduce the deficiency of these vitamins in the country.

In Ethiopia, soybean oil is one of the most commonly used edible oils due to its affordability and wide availability (FAO 2021). Its high fat content makes it an ideal carrier for fat‐soluble vitamins like vitamin A (retinyl palmitate) and vitamin D_3_ (cholecalciferol), promoting better absorption in the body. However, the success of oil fortification efforts depends heavily on the stability of these vitamins during storage and the typical cooking methods used. Both vitamins A and D_3_ are sensitive to external factors such as heat, light, oxygen, and moisture, which can cause them to degrade over time (Favaro et al. 2020). Traditional Ethiopian cooking practices—such as boiling, frying, and sautéing at high temperatures—can significantly reduce the levels of these fortified vitamins. For example, popular dishes like *doro wat* and *shiro wat* involve long cooking times and intense heat conditions (180–250) that may exacerbate vitamin losses. Furthermore, local storage environments, often marked by high ambient temperatures, exposure to light, and limited access to advanced packaging materials, may further accelerate vitamin degradation during the shelf life of fortified oils (Ramakrishnan and Devi 2018).

Evidence from other low‐resource settings suggests that losses of vitamin A during cooking and storage can be substantial. Some studies have reported that as much as 60% of vitamin A content can be lost during deep‐frying or extended storage under inadequate conditions (Fiedler and Afidra 2010). Similarly, vitamin D_3_ is vulnerable to oxidative breakdown, especially in oils exposed to harsh storage environments (Hemery et al. 2015). Despite the expansion of fortification programs worldwide, there is still a lack of detailed information about how fortified oils perform under Ethiopian conditions—both during traditional cooking and in everyday storage settings. Without localized data, it is difficult to accurately assess the true impact of fortification efforts on public health. Understanding the extent of vitamin loss is critical to ensuring that fortified products provide the intended nutritional benefits to vulnerable communities. This study, therefore, seeks to investigate the retention of vitamins A and D_3_ in fortified soybean oil during traditional Ethiopian cooking processes and to assess the stability of these vitamins during storage.

## 2 Materials and Methods

### 2.1 Sample Collection and Technique

Refined, bleached, and deodorized soybean oil (RBDSBO) was sourced from Kunifira Agro‐Processing PLC in Addis Ababa, Ethiopia. On the first day of production, four 1‐L samples were randomly selected from different production batches. These included one sample for assessing the retention of vitamins A and D_3_ during cooking, two for evaluating shelf‐life stability over time, and one designated as a control. Commercially prepared vitamin A (retinyl palmitate) and vitamin D_3_ (cholecalciferol) were obtained from the Global Alliance for Improved Nutrition (GAIN), while all analytical standards were purchased from a local chemical supplier. The collected oil samples were combined in a clean bowl prior to treatment. One sample was sealed in a PET container before the vitamin premix was added, while the remaining three were fortified and then sealed.

The final samples—labeled Oil 1 through Oil 4—were stored in the laboratory for subsequent analysis. Oil 1 served as the control to measure baseline levels of vitamins A and D_3_ and to assess oil quality prior to fortification. Oil 2 was designated for retention studies during cooking, while Oil 3 and Oil 4 were allocated for shelf‐life stability testing during storage.

### 2.2 Chemical Analysis of the Oils

The chemical parameters of the oils were analyzed before and after fortification in the initial, 3^rd^, and 6^th^ months of storage.

#### 2.2.1 Determination of Moisture and Volatile Matter Content

To determine the moisture content and volatile matter in the oil samples, we used a GEN LAB‐OV/125/SS/FDIG/A oven from the UK, following the ISO 662:2016 standard. In short, a clean glass vessel was first dried in an oven at 103°C ± 2°C for 30 min, and then allowed to cool in a desiccator before being weighed. Next, 5 g of the oil sample was placed into the dried vessel, which was then heated in the oven at the same temperature for 1 h. After cooling to room temperature in a desiccator, the vessel was weighed again; this step was repeated until the weight stabilized. Finally, the percentage of moisture and volatile matter was calculated using the standard formula. Moisture content (%) $=\frac{M1-M2}{M1-M0}\times 100\%$, where *M*_*0*_ denotes the weight in gram of the glass vessel, *M*_*1*_ denotes the weight in gram of the sample and the glass vessel, and *M*_*2*_ denotes the weight in gram of the sample and residue.

#### 2.2.2 Determination of Peroxide Value

The peroxide values of the oil samples (meq O_2_/kg) were determined using an official method of AOCS Cd 8‐53 with slight modification. Based on the expected peroxide value of soybean oil, 5 g±0.1 g of oil was weighed into a 250‐mL Erlenmeyer flask. The sample was dissolved in 30 mL of glacial acetic acid–chloroform solution (3:2). After the addition of 0.5 mL of saturated potassium iodide solution, the mixture was kept in the dark at ambient temperature for 1 min. After the addition of 30 mL of distilled water, 2 mL of the saturated starch solution was added, and the mixture had changed from dark purple to dark brown. The solution was titrated against Na_2_S_2_O_3_ (0.01 N) until the mixture was changed into a white color. A blank determination was conducted, and the concentration of peroxide value was calculated using the following: formula.

$$\mathrm{PV}=\frac{\left(\;\mathrm{Vs}-\mathrm{Vb}\right)\times N\times 1000}{W}$$

where, *V*_s_ represents the volume of Na_2_S_2_O_3_, *V*_b_ represents the volume of Na_2_S_2_O_3_ used in the blank samples, *N* represents the normality of Na_2_S_2_O_3_ (meq/mL used for titration), and *W* represents the weight of the cooking oil sample (g).

#### 2.2.3 Determination of Iodine Value

The official method of AOAC (2000) was employed to determine the iodine value of oil samples. Exactly 0.25 g of oil was weighed into a 250‐mL conical flask and dissolved in 10 mL of chloroform and 30 mL of Hanus iodine solution and allowed to stand in the dark for 30 min with occasional shaking. Then, 10 mL of 15% potassium iodide was added and shaken thoroughly, and 100 mL of freshly boiled and cooled water was added to wash down any free iodine from the stopper. The final solution was titrated against Na_2_S_2_O_3_ (0.1 N) until the yellow color was formed. Then, 2–3 drops of the starch indicator were added, and the mixture changed to blue and was titrated until the blue color completely disappeared. Then, a blank determination was conducted, and the concentration was calculated using the following formula.

$$\text{Iodine value}=\frac{\left(B-S\right)\times N\times 0.127g/\mathrm{meq}\times 100}{W}$$

where *B* represents the volume of Na_2_S_2_O_3_ for blank, *S* represents the volume of Na_2_S_2_O_3_ for sample, and *N* represents the normality of Na_2_S_2_O_3_ and *W* = the weight of the sample.

#### 2.2.4 Determination of Acid Value

An official method of AOCS (2003) Ca 5a‐40 was employed to measure the acid value of the oil sample. The oil was mixed well before weighing. The mass of the test sample was selected based on the expected acid value. According to WFP (2011) soybean oil specification, the acid value must be below 0.6 mg maximum of KOH/g oil, which means less than 1 g. Accordingly, 20 g of oil sample was weighed into a 250 mL conical flask. About 50 mL of 95% ethanol containing 0.5 mL phenolphthalein (1%) was boiled at 70°C and added to the flask. Then, the mixture was titrated against 0.1 N potassium hydroxide and shaken vigorously until it changed from colorless to light pink color. A blank determination was conducted, and the amount of acid value was calculated using the formula as follows:

$$\text{Acid value}=\frac{56.1\times V\times N}{M}$$

where *V* represents the volume (in mL) of standard potassium hydroxide, *N* represents the normality of potassium hydroxide, and *M* represents mass of test portion in g.

#### 2.2.5 Determination of Free Fatty Acid

The free fatty acid (FFA) value of the oil sample was measured using an official method of AOCS (2003) Ca 5a‐40. The oil was mixed well before weighing. The mass of the test sample was taken based on the expected acid value. According to WFP (2011) soybean oil specification, the acid value must be below 0.6 mg maximum of KOH/g oil, which means less than 1 g. Accordingly, 20 g of oil sample was weighed into a 250‐mL conical flask. About 50 mL of 95% ethanol containing 0.5 mL phenolphthalein (1%) was boiled at 70°C and added to the flask. Then, the mixture was titrated against 0.1 N potassium hydroxide and shaken vigorously until it changed from colorless to light pink color. A blank determination was conducted, and the amount of FFA was calculated using the formula written below.

Free fatty acid content (%) = $\frac{V\times N\times M}{10\times m}$, where *V* represents the volume (in mL) of the standard volumetric solution of potassium hydroxide, *N* represents the normality of potassium hydroxide, *M* represents the molar mass in (g per mole) of the acid chosen from Table 2, and *m* represents the mass of the test portion.

### 2.3 Fortification of Soybean Oil With Vitamins A and D_3_

The vitamin premix, containing 1,000,000 IU/g of vitamin A (retinol palmitate) and 100,000 IU/g of vitamin D_3_ (cholecalciferol) stabilized with vitamin E (alpha‐tocopherol) purchased from GAIN, was added to RBDSBO. Six hundred milligrams of vitamin premix was added to 3 L of oil and homogenized using a general laboratory homogenizer Omni International GLH‐115, USA, in the dark at room temperature for about 30 min. The oil was then blown with nitrogen gas and sealed in the PET bottle (Hemery et al. 2015).

#### 2.3.1 Storage of Fortified Oil Samples

Oil 2 was kept at room temperature and used to test how well vitamins A and D_3_ were retained during cooking on the first day of storage. Oils 3 and 4 were stored for 6 months—Oil 3 at room temperature and Oil 4 at 37°C—to evaluate their shelf life. The average room temperature in Addis Ababa, around 25°C, reflects the cooler parts of Ethiopia's climate, while 37°C was chosen to simulate conditions in the country's hotter regions.

#### 2.3.2 Quantification of Vitamins A and D_3_ in Fortified Soybean Oil Before Cooking

The amount of vitamins A and D_3_ of the oil was quantified in the first week of fortification using a HPLC UV–visible detector (2489 UV/Vis Detector manufactured by Shimadzu Corporation) as described in the AOAC Official Method (2001.13) and AOAC Method (2002.05) with slight modifications.

The quantification was carried out in Bless Agri Food Laboratory in Addis Ababa, Ethiopia.

#### 2.3.3 Sample Saponification and Extraction

Five grams of oil sample and standards were weighed appropriately into flasks, upon which 40 mL of 95% ethanol and 50 mg of pyrogallic acid were added to each sample and standard. This was followed by the addition of 10 mL of 50% KOH to each flask and standard. Then, the whole content was placed in a water bath at 80°C for 45 min. The flasks were then cooled to room temperature (25°C) using cooled water, and 10 mL of glacial acetic acid was added to each flask to neutralize KOH. Samples and standard were extracted five times with hexane; the pooled extracts were then washed with 50 mL of water until the aqueous layer appeared colorless when adding 2–3 drops of phenolphthalein. Then, the mixture was filtered through a 0.45‐μmMSÒNylon membrane filter and concentrated to dryness using a rotary evaporator under nitrogen gas at 40°C. The extract was reconstituted in 10 mL of pure methanol for vitamin A and in methanol–acetonitrile (4:1) for vitamin D_3_ and passed through a 0.45‐μmChromafilXtrafilter before HPLC injection (AOAC Official Method (2001.13) and AOAC Method (2002.05)).

#### 2.3.4 Chromatographic Determination of Vitamin A

**FIGURE 1:**
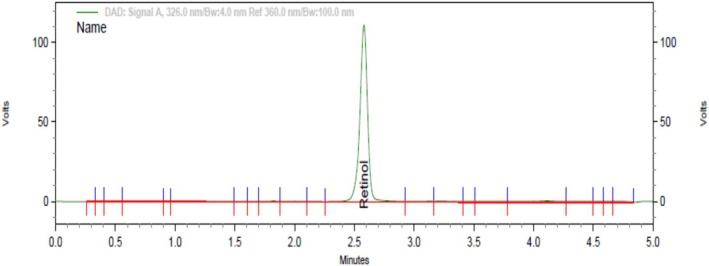
Chromatogram of vitamin A determination in the oil sample.

The HPLC system was started and allowed to warm up and equilibrate with a mobile phase flow rate of 1 mL/min. A ratio of 97.5:2.5 (methanol and water) mobile phase composition and reversed‐phase C18 column was used. Then, the vitamin A standard that has been saponified along with the sample was injected into HPLC, and the mobile phase was adjusted to achieve a resolution of 1.5. Then, the sample was injected into the HPLC UV–visible detector, and retinol was determined at a wavelength of 326 nm and 2.5 min retention time. Finally, the concentration of vitamin A in the sample was calculated by the following formula (AOAC Official Method (2001.13)) (Figure 1).

Vitamin A mg/kg (as retinol) = $\frac{A}{W}\times 10$, where *A* represents the total test sample peak area of all *trans* and 13‐*cis* retinol, 10 represents the dilution factor of the test portion, and *W* represents weight of test portion (g).

#### 2.3.5 Chromatographic Determination of Vitamin D_3_

**FIGURE 2:**
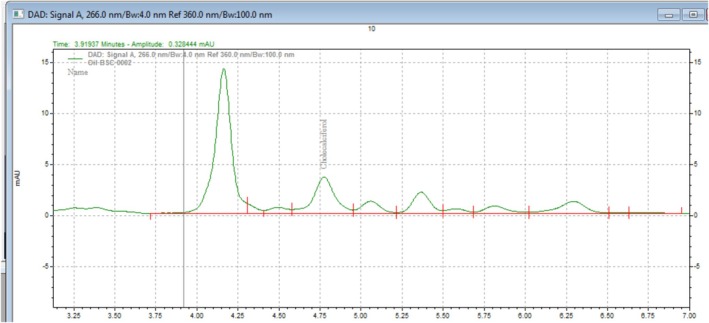
Chromatogram of vitamin D_3_ determination in oil sample.

The HPLC system was started and allowed to warm up and equilibrate with a mobile phase flow rate of 1 mL/min. A ratio of 97.5:2.5 methanol–water mobile phase composition and reversed‐phase C18 column was used for quantitative analysis. The stability of the retention time was checked by injecting the working standard solution, and the retention time was flocculated to less than ±1%. The sample was injected into the HPLC UV–visible detector, and vitamin D_3_ was determined at a wavelength of 266 nm and a retention time of 4.5 min. Finally, the concentration of vitamin D_3_ in the oil sample was calculated using the following formula (AOAC Method (2002.05)) (Figure 2).

$$\text{Vitamin}\;D_{3}\;\mathrm{mg}/\mathrm{kg}=\frac{\mathrm{AD}3}{W}\times 10$$

where AD_3_ represents the peak area of vitamin D_3_, *W* represents the weight of the test portion, and 10 represents the dilution factor of test portion.

#### 2.3.6 Quantification of Vitamins A and D_3_ in Cooked Stew

##### 2.3.6.1 Stew Preparation

The most common type of stew in Ethiopia (Shiro) was prepared following the same commonly used traditional way of cooking. The entire ingredients (chickpea flour, onion, garlic, tomato, pepper powder, salt, chili, and water) used for the preparation of the stew were weighed before cooking, and the mixture also was weighed after cooking to know the mass fraction of the oil in the mixture. A known amount of oil was used to prepare the stew by heating at a temperature of 250°C; the entire ingredients (250 g chickpea flour, 15 g onion, 8 g garlic, 12 g tomato, 4 g pepper powder, 3 g salt, 2 g chili and 1000 mL water) were added, and the mixture was heated on a standard laboratory hot plate. During cooking, the time and temperature were recorded. The whole content was cooked for about 1 h and 20 min at 250°C until the color of the mixture changed to the color of the traditional stew (shiro).

##### 2.3.6.2 Saponification and Extraction

Five grams of the stew sample and standard was weighed appropriately into flasks; 40 mL of 95% ethanol and 50 mg of pyrogallic acid were added to the sample and standard. Then, 10 mL of 50% KOH was added to each flask and standard and placed in a water bath at 80°C for 45 min. The flasks were cooled to room temperature using cooled water, and 10 mL of glacial acetic acid was added to each flask to neutralize KOH. The sample and standard were extracted five times with hexane. The pooled extracts were then washed with 50 mL of water until the aqueous layer appeared colorless upon the addition of 2–3 phenolphthalein drops. Then, the mixture was filtered through a 0.45‐μmMSÒNylon membrane filter and concentrated to dryness using a rotary evaporator under nitrogen gas at 40°C. The extracts were reconstituted in 10 mL of pure methanol for vitamin A and in methanol–acetonitrile (4:1) for vitamin D_3_ and passed through a 0.45‐μm ChromafilXtrafilter before HPLC injection (AOAC Official Method (2001.13) and AOAC Method (2002.05)).

##### 2.3.6.3 Chromatographic Determination of Vitamin A

The HPLC system was started and allowed to warm up and equilibrate with mobile phase at a flow rate of 1 mL/min. Then, standard vitamin A that has been saponified along with the sample was injected into the HPLC system, and the mobile phase was adjusted to achieve a resolution of 1.5 or better for *cis* and *trans* forms. Then, the sample was injected into the HPLC UV–visible detector, and retinol was determined at a wavelength of 326 nm and with a retention time of 2.5 min. Finally, the concentration of vitamin A in the stew sample was calculated by the following formula (AOAC Official Method (2001.13)).

Vitamin A mg/kg (as retinol) = $\frac{A}{W}\times 10$, where, *A* represents the total test sample peak area of all *trans* and 13‐*cis* retinol, 10 represents the dilution factor of test portion, and *W* represents the weight of test portion (in g).

To obtain the concentration of vitamin A in the oil, the mass fraction of the oil was calculated by dividing the mass of oil to the total mass of the mixture and multiplied by the weight of the test portion. Then the concentration of vitamin A in the oil was calculated using the following formula. Vitamin A mg/kg (as retinol) = $\;\frac{W\times C}{\mathrm{Mf}}$.where *W* represents the mass of the test portion (in g), *C* represents the concentration of vitamin in the mixture in ppm, and Mf represents the mass fraction of the oil (in g).

##### 2.3.6.4 Chromatographic Determination of Vitamin D_3_

The HPLC system was started and allowed to warm up and equilibrated with a mobile phase flow rate of 1 mL/min. A ratio of 4:1 methanol–acetonitrile (v/v) mobile phase composition was prepared for quantitative analysis. The stability of the retention time was checked by injecting the working standard solution, and the retention time was flocculated to less than ±1%. The sample was injected into the HPLC UV–visible detector, and vitamin D_3_ was determined at a wavelength of 266 nm and the retention time of 4.5 min. Finally, the concentration of vitamin D_3_ in the oil sample was calculated using the following formula AOAC Method (2002.05).

$$\text{Vitamin}\;D_{3}\;\mathrm{mg}/\mathrm{kg}=\frac{\mathrm{AD}3}{W}\times 10$$

where AD_3_ represents the peak area of vitamin D_3_, *W* represents the weight of the test portion and 10 represents the dilution factor of test portion.

To obtain the concentration of vitamin D3 in the oil, the mass fraction of the oil was calculated by dividing the mass of oil by the total mass of the mixture and multiplied by the weight of the test portion. Then the concentration of vitamin D_3_ in the oil was calculated using the following formula.

$$\text{Vitamin}\;D_{3}\;\mathrm{mg}/\mathrm{kg}=\frac{W\times C}{\mathrm{Mf}}$$

where *W* represents the mass of the test portion (in g), *C* represents the concentration of vitamin D_3_ on the mixture in ppm, and Mf represents the a mass fraction of the oil (in g).

#### 2.3.7 Shelf‐Life Stability Testing of Vitamins A and D_3_ in Fortified Soybean Oil During Storage

The shelf life of oil was determined using normal stability life testing (NSLT). Oil 3 stored at room temperature (25°C) and Oil 4 (stored at 37°C) were stored for 6 months. The vitamin A and vitamin D_3_ content were analyzed in the initial, 3, and 6 months. The sample preparation and chromatographic determination were carried out following the same mentioned procedure described above. The percentage of vitamin A and vitamin D_3_ retention was calculated at the 3 and 6 months of storage using the following formula (Hemery et al. 2015).

$$\%\text{of retention}=\frac{\left(\text{vitamin}\right)t}{\left(\text{vitamin}\right)0}\times 100$$

where (vitamin)^0^ represents the initial content of vitamin and (vitamin)^t^ represents the final content of vitamin.

$$\%\text{retention vitamin}\;A=\frac{39.25}{65.78}\times 100=60.04\%$$

$$\%\text{retention vitamin}\;D_{3}=\frac{4.1}{4.21}\times 100=97.39\%$$

### 2.4 Experimental Design and Statistical Analysis

The experimental design used in this study was a completely randomized design (CRD) and data were analyzed by analysis of variance (ANOVA) using statistical software SPSS version 20, and significant differences were tested at (*p* < 0.05). All laboratory analyses were performed in triplicate, and averages were presented.

## 3 Result and Discussion

### 3.1 Chemical Characteristics of Soybean Oil Before and After Fortification

The chemical characteristics of the soybean oil before and after fortification is reported in Table 1. There was no significant difference in all of the chemical parameters except FFA value before and after fortification. The shelf‐life stability of fortified vitamin in the oil depends upon the shelf‐life stability of the oil, which depends on the chemical characteristics of the oil like peroxide value, acid value, moisture, iodine value, and level of FFA. For instance, low moisture content in the oil is advantageous in terms of storage stability (Orhevba and Efomah 2012). With this regard, the percentage moisture and volatile matter in both fortified and unfortified soybean oils in this study were 0.1%, which is below the set maximum limit (i.e., < 0.2%) by World Food Program (2011).

**TABLE 1:** Chemical properties of soybean oil before and after fortification at initial storage period.

Parameters	Type of soybean oil	
	Unfortified oil	Fortified oil
Moisture and volatile mater (%)	0.10 ± 0.01^a^	0.10 ± 0.01 ^a^
Peroxide value (meqO_2_/kg oil)	1.00 ± 0.09 ^a^	1.20 ± 0.09 ^a^
Iodine value (g/100 g oil)	118.00 ± 2.30 ^a^	118.00 ± 3.05 ^a^
Free fatty acid (%)	0.087 ± 0.001^b^	0.098 ± 0.001^a^
Acid value (mg KOH/g oil)	0.17 ± 0.02^b^	0.20 ± 0.06^a^

*Note:* Data are expressed as mean ± SE. All analyses were done in triplicate. Mean values in the same row with different superscripts are statistically significant at *p* < 0.05.

Similarly, peroxide value (PV) is used as an indicator of the deterioration of edible oils. In this study, the peroxide value of the unfortified and fortified soybean oils was 1.0 and 1.2 meqO_2_/kg, respectively (Table 1). Fresh oils have a PV below 10 meqO_2_/kg (Vidrih et al. 2010). According to the recommendation by WFP (2011), the maximum PV of fresh refined and fortified soybean oils must not exceed 2 meqO_2_/kg. Also, as per Ethiopian standards, edible oils should contain a PV below 10 meqO_2_/kg oil (Compulsory Ethiopian Standard CES‐16, 2014; Compulsory Ethiopian Standard CES‐19, 2014). Iodine value (IV), which indicates the degree of unsaturation in oils, was found to be 118 g of iodine absorbed/100 g of oil in both fortified and unfortified soybean oils (Table 1). The higher the IV, the more unsaturated the fatty acid composition of fat and/or oil (Fakhri and Qadir 2011; Scrimgeours 2005).

Another key shelf life–determining factor of oil is the level of FFA. Oils and fats are well characterized/distinguished mainly by their fatty acid composition. The levels of FFA in unfortified and fortified oils were 0.087 and 0.098, respectively (Table 1). According to WFP (2011), the maximum FFA (%) in fresh refined and fortified soybean oils must be 0.1%. As reported in Table 1, the chemical characteristics of the soybean oil before and after fortification satisfied the set requirements of both WFP and ESA. As a result, this study indicated that fortification had no effect on the chemical parameter of oil.

### 3.2 Retention of Vitamins A and D_3_ During Cooking

The chromatographic method for determination of vitamins A and D_3_ was validated ahead of the sample analysis. In brief, the LOD for the vitamins A and D_3_ determinations with S/*N* ≥ 3 was 1.83 and 0.09, respectively. The LOQ for vitamins A and vitamin D_3_ was 5.5 and 0.27 μg/mL, respectively, with S/*N* ≥ 10. The precision of the method was evaluated through the repeatability of the method by assessing 10 replicate injections of vitamin standard at the same concentration during the same day under the same experimental conditions to obtain an acceptable %RSD. The % RSD for peak area of vitamins A and D_3_ was 4.5 and 5.2, respectively. Standard curves were prepared for both vitamins by using the standard solutions covering the concentration range from 5 to 60 μg/mL for vitamin A and 0.15 to 3.6 μg/mL for vitamin D_3_. Peak areas of the different vitamins were plotted against the concentrations and linear regression analysis (unweighted; regression line forced through zero) was used to calculate the equation and the correlation coefficient of the standard curves. The recovery was assessed by spiking the oil with standard solutions. Following this, the recovery rate was determined for the added quantity of standard and the mean recovery rate was recorded as 100% and 100.06% for vitamins A and D_3_, respectively. The specificity/selectivity of the method was assessed by the visual inspection and comparison of chromatograms of blank samples and samples containing different amounts of vitamins (CRM, PT, and spiked samples). The linear working range was found to be between 5 and 60 μg/mL for vitamin A and between 0.15 and 3.6 μg/mL for vitamin D_3_.

Table 2 shows the concentration of vitamins A and D_3_ in fresh unfortified, fresh fortified, and fortified cooked soybean oils. There was a significant difference in vitamin A content among the three oil samples (*p* < 0.05). Since vitamin A (retinol) is not found in plant‐based foods, the fresh soybean oil had no vitamin A. After fortification, the vitamin A content in the fresh oil was 65.78 mg/kg. At the same time, after the fortified oil was used to cook Ethiopian traditional stew (Shiro) at 250°C for 1 h and 20 min, the vitamin A concentration was reduced to 39.25 mg/kg. Apparently, this reduction might be due to the high‐temperature processing for a prolonged time. Vitamin A is quite stable during boiling, simmering, and stewing (100°C–120°C), even when heated over an extended period (Favaro et al. 1991). Favaro et al. (1991) found that 99% of vitamin A was retained when fortified oil was added to rice and cooked for 15 min at 100°C–120°C. The same authors reported that 88% of vitamin A was retained when oil was added to beans and boiled for 90 min at 100°C–120°C. In this study, the oil was added to Shiro (commonly made of peas and spices) and boiled at 250°C for 1 h and 20 min. At this cooking time and temperature, 60.04% of vitamin A was retained. A previous study also reported that 64% of vitamin A was retained when the soybean oil, corn oil, and safflower oil were added to different foods and cooked at more than 200°C for 30 min.

**TABLE 2:** Concentration of vitamins A and D_3_ in unfortified fresh, fortified fresh, and fortified cooked soybean oils (mg/kg).

Type of soybean oil	Concentration of vitamins (mg/kg)	
	Vitamin A	Vitamin D_3_
Unfortified fresh	0.00 ± 0.00^c^	0.00 ± 0.00^b^
Fortified fresh	65.78 ± 0.01^a^	4.21 ± 0.01^a^
Fortified cooked	39.25 ± 0.01^b^	4.10 ± 0.01^a^

*Note:* Data are expressed as mean ± SE, all analyses were done in triplicate. Mean values in the same column with different superscript are significantly different at *p* < 0.05.

However, there was no significant difference in vitamin D_3_ content between fresh and cooked oils because vitamin D_3_ is less sensitive to heat and light (Favaro et al. 1991). There was a slight loss of vitamin D_3_ during cooking which dropped from 4.21 to 4.10 mg/kg but, scientifically, if mean values in the same column/row are with the same superscript, there is no significant difference. Many researchers reported that both vitamin D_2_ and vitamin D_3_ are moderately stable in many foods during storage and retain better on moderate heat processing. A study in Denmark concluded that no significant difference was found between the retention of vitamin‐D_3_– and vitamin‐D_2_–spiked sunflower oil before and after household cooking. In the present study, there was also no significant difference in vitamin D_3_ concentration before and after household cooking. Another reason for the acceptable retention of both vitamins A and D_3_ in cooked food is that it is due to the presence of antioxidants. Some studies have documented the use of antioxidants to enhance the stability of vitamin A in the oil (Favaro et al. 1991). Steenhoek (1997) tested the stability of vitamin A with and without BHA in Indonesian vegetable oil during double frying conditions at temperatures up to 193°C. In the first test with 200 ppm of BHA, 77% vitamin A was retained. In the second test without BHA, 37% of vitamin A was retained. Gopal and Ketyum (1956) also found that after 5 min of frying in Vanaspati (vegetable ghee) at 200°C, the retention of vitamin A was 71% with BHA and 60% without BHA. The gap between the two studies stated above was due to frying time. In our study, vitamin E (alpha‐tocopherol) was added to soybean oil as an antioxidant and the cooking temperature was 250°C and time were 1 h and 20 min. In this scenario, more than 60% of vitamin A was retained.

So the gap with the previous study could be due to the cooking time, temperature, types of oil, and the type of antioxidant. Steenhoek and Gospal used the antioxidant BHA, Indonesian oil, and Vanaspati for their experiment. Several sources suggested that TBHQ and BHA might offer superior protection, particularly in soybean oil (Bauernfeind 1953). The retention of vitamins A and D_3_ during cooking on fortified soybean oil has been studied in other countries, but not yet in Ethiopia. Traditional cooking practice in Ethiopia is quite different from the cooking and food preparation of other countries (Ramakrishnan and Devi 2018). As explained above, most of the Ethiopian mothers have developed a culture of cooking the food at high temperatures for a long time to make the food delicious, which harms the retention of heat‐sensitive nutrients, mostly vitamins (Ramakrishnan and Devi 2018). Also, most of the Ethiopian foods are very spicy and ingredient‐rich, which have an effect on vitamin retention during cooking. Having this postulation, we were inspired to evaluate the retention of vitamins A and D_3_ in fortified soybean oil during Ethiopian traditional cooking style to provide concrete input to edible oil producers, government stakeholders, and society.

### 3.3 Chemical Characteristics of Fortified Soybean Oil After 3 Months of Storage at Room Temperature and 37°C

The chemical characteristics of unfortified fresh, fortified fresh, and fortified oils stored at room temperature and at 37**°**C after 3 months of storage is reported in Table 3. The PV and FFA level had significantly increased during storage, while no significant change was observed in moisture content and acid value. Even though peroxide was increased during storage, it was an acceptable value as recommended by WFP (2011) (i.e., < 10 meqO_2_/kg). Similarly, the FFA level of the fortified soybean oil significantly increased during the third month of storage.

**TABLE 3:** Chemical properties of unfortified fresh, fortified fresh, and fortified soyabean oils stored at room temperature and at 37°C after 3 months of storage.

Parameters	Type of soybean oil			
	Oil 1	Oil 2	Oil 3	Oil 4
Moisture and volatile mater	0.10 ± 0.01^a^	0.10 ± 0.01 ^a^	0.10 ± 0.02 ^a^	0.20 ± 0.06 ^a^
Peroxide value (meqO_2_/kg oil)	1.00 ± 0.09^c^	1.20 ± 0.09^c^	4.17 ± 0.02^b^	5.50 ± 0.25^a^
Free fatty acid (%)	0.09 ± 0.00^b^	0.10 ± 0.00^b^	1.13 ± 0.01^ab^	1.15 ± 0.02^a^
Acid value (mg KOH/goil)	0.17 ± 0.02 ^a^	0.20 ± 0.06 ^a^	0.27 ± 0.01 ^a^	0.30 ± 0.12 ^a^

*Note:* Data are expressed as mean ± SE. All analyses were done in triplicate. Mean values in the same row with different superscripts are significantly different at *p* < 0.05. Oil 1‐control (unfortified oil); Oil 2: fortified fresh oil; Oil 3: fortified oil stored at room temperature; Oil 4: fortified oil stored at 37°C.

There was also a significant difference in PV between the fortified oil stored at room temperature and 37°C; apparently, the latter had a higher PV. The stability of vitamins in oil depends upon the physicochemical characteristics, storage conditions, and the packaging material of the oil. One of the important parameters used to assess the quality of soybean oil is the peroxide value, which is an indicator of the level of lipid oxidation (Codex Alimentarius—Food labeling—Complete texts 2013). According to the recommendation of Codex 210, Codex Alimentarius—Food labeling—Complete texts (2013) and WFP (2011) the PV of fortified soybean oil must not exceed the upper limit (10 meq O_2_/kg) during storage.

In the present study, the PV had increased with storage time and temperature. Another key parameter of oil quality assessment is FFA value, which should be < 1.15% in fortified soybean oil as per World Food Program (2011) recommendation. The FFA% in all the oil samples in the present study was within the standard limit. However, as compared to the value at the first month of storage, there was significant increment on third months of storage. Moreover, the increment was also observed as the storage temperature increased. In fact, increased PV and FFA in edible oils with storage temperature and time were supported by previous studies too (Andarwulan et al. 2014).

### 3.4 Shelf‐Life Stability of the Vitamins A and D_3_ During 3 Months of Storage

The vitamin A and D_3_ contents of fortified soybean oil stored at different storage temperatures after 3 months of storage are reported in Table 4 (*p* < 0.05). Accordingly, the vitamin A content in the oil had significantly decreased after 3 months of storage In fact, the vitamin A content was more reduced in the oil stored at 37°C. The amount of vitamin A loss stored at room temperature and 37 degree celcius was 4.36% and 4.42%, respectively. Favaro et al. (1991) reported that, in sealed and opaque containers, losses of vitamin A are negligible for a year Similarly, Kolanowski and Berger (1999) reported that vitamin A in soybean oil stored at 20°C–25°C retained 95%–100% of its original content over 3 months. Hence, keeping fortified soybean oil in a cool place is better to reduce the possible loss of vitamin A due to exposure to temperature. Also, incorporation of antioxidants in fortified oils will protect against the loss of vitamins during storage (Favaro et al. 1991). For instance, with BHA, the stability of vitamin A in fortified soybean oil was 100‐93% after 3 months of storage at 32°C (Favaro et al. 1991). This is a similar finding to the present study, in which 96% of vitamin A was retained after 3 months of storage at both room temperature and 37°C. In the present study, vitamin E was added to the oil as an antioxidant.

**TABLE 4:** Concentration of vitamins A and D_3_ (mg/kg) of fresh fortified and unfortified soybean oils stored at room temperature and at 37°C for 3 months.

Type of soybean oil	Concentration of vitamins (mg/kg)	
	Vitamin A	Vitamin D_3_
Fresh, unfortified oil	0.00 ± 0.00^d^	0.00 ± 0.00^c^
Fresh, fortified oil	65.78 ± 0.01^a^	4.21 ± 0.01^a^
Fortified oil stored at RT	62.91 ± 0.0b^b^	1.99 ± 0.01^b^
Fortified oil stored at 37°C	62.87 ± 0.01^c^	1.96 ± 0.02^b^

*Note:* Data are expressed as mean ± SE, all analyses were done in triplicate. Mean values in the same column with different superscript are significantly different at *p* < 0.05. Oil 1: control (unfortified oil); Oil 2: fortified fresh oil; Oil 3: fortified oil stored at room temperature; Oil 4: fortified oil stored at 37°C.

As reported in Table 4, the vitamin D_3_ content in the oil had significantly decreased after a storage period of 3 months (*p* < 0.05). At both storage temperatures, the loss was around 50%. Apparently, the more pronounced reduction during storage was mainly due to storage time. Many researchers reported that both vitamin D_2_ and vitamin D_3_ are moderately stable in many foods during storage. Kolanowski and Berger (1999) reported the reduction of vitamin D_2_ and D_3_ in oil and fat with increasing storage time. As compared to vitamin A, vitamin D_3_ highly decreased during storage. This indicated higher stability of vitamin A than vitamin D_3_ during storage.

Commonly, soybean oil in developed countries is produced from genetically modified seeds. Previous studies indicated that soybean oil produced from genetically modified seed has a longer shelf life than oil produced from organic seed. This is because the genetically modified soybean seeds have less FFA composition (low linoleic acid and linolenic acid) than organic soybean. Neff and List (1999) investigated how soybean lines that were genetically modified for high C16:0 and high C18:0 changed the oxidative stability of natural oils. The stability of vitamins is dependent on the stability of the oil; if the oil is oxidized, the vitamin concentration will be reduced upon storage (Morales and Przybylski, 2013). Soybean oil in Ethiopia is produced from natural (organic) soya seed. Yet, to our knowledge, there was no study on the shelf stability of soybean oil in Ethiopia. As indicated in Table 4, the retention (%) of both vitamins A and D_3_ after 3 months of storage was 95.64% and 47.27% at room temperature and 95.58% and 46.56% at 37°C, respectively. At both storage temperatures, the highest retention was observed in vitamin A concentration.

### 3.5 Chemical Characteristics of the Oil During 6 Months of Storage

As reported in Table 5, there was a significant difference in the chemical characteristics of fortified soybean oil during the 6 months of the storage period. The moisture/volatile matter content, PV, and FFA had increased during the 6 months of storage significantly. Moreover, the higher storage temperature (37°C) had an obvious effect on deteriorating the quality of the oil as compared to room temperature storage. However, although the values had an increasing trend, the PV and AV were within the acceptable limit during the six months of storage. However, the values of moisture and FFA in Oil 4 were unacceptable (i.e., 0.25% and 2% respectively). FFAs are produced by the hydrolysis of oils and fats. The level of FFA depends on time, temperature, and moisture content in the oil. As FFA is less stable in neutral oil, they are more prone to oxidation and rancidity. Thus, FFA is a key feature linked with the quality and commercial value of oils and fats. The remarkable rise of FFA in the fortified soybean oil stored at 37°C might be linked with the increased moisture content. This might have accelerated the hydrolysis process in the oil (Nielsen 1994).

**TABLE 5:** Chemical properties of unfortified fresh, fortified fresh, fortified soybean oils stored at room temperature and at 37°C after 6 months of storage.

Parameters	Type of soybean oil			
	Oil 1	Oil 2	Oil 3	Oil 4
Moisture and volatile mater	0.10 ± 0.01^c^	0.10 ± 0.01^c^	0.15 ± 0.01^b^	0.25 ± 0.02^a^
Peroxide value (meqO_2_/kg oil)	1.00 ± 0.09^c^	1.20 ± 0.09^c^	5.17 ± 0.01^b^	7.00 ± 0.04^a^
Free fatty acid value (%)	0.09 ± 0.00^c^	0.10 ± 0.01^c^	1.15 ± 0.02^b^	2.00 ± 0.01^a^
Acid value (mg KOH/goil)	0.17 ± 0.02^b^	0.20 ± 0.06^b^	0.30 ± 0.11^b^	0.40 ± 0.02^ab^

*Note:* Data are expressed as mean ± SE. All analyses were done in triplicate. Mean values in the same row with different superscripts are significantly different at *p* < 0.05. Oil 1: control (unfortified oil); Oil 2: fortified fresh oil; Oil 3: fortified oil stored at room temperature; Oil 4: fortified oil stored at 37°C.

### 3.6 Shelf‐Life Stability of the Vitamins A and D_3_ During 6 Months of Storage

In this study, at 6 months of storage, the vitamin A concentration in the oil had decreased significantly (*p* < 0.05). Also, at 37°C storage temperature, the loss was significantly higher than room temperature storage (Table 6). Briefly, the vitamin loss in the fortified oil stored at room temperature and 37°C was 5.42% and 8.77%, respectively. Similarly, a minimal/negligible loss of vitamin A in fortified oil kept in sealed and opaque containers that protected light and air for a year was reported by Favaro et al. (1991). In the present study, the fortified soybean oil was sealed in PET packaging and stored at room temperature and 37°C for 6 months retained 94.58% and 91.23% vitamin A, respectively. Kolanowski and Berger (1999) reported retention of vitamin A in margarine to be 90%–95% and 85%–90% after 3 months and 6 months of storage, respectively, in a sealed can container at 25°C.

**TABLE 6:** Concentration of vitamins A and D_3_ (mg/kg) in fresh fortified and unfortified soybean oils stored at room temperature and at 37°C for 6 months.

Type of soybean oil	Concentration of vitamins (mg/kg)	
	Vitamin A	Vitamin D_3_
Fresh, unfortified oil	0.00 ± 0.00^d^	0.00 ± 0.00^d^
Fresh, fortified oil	65.78 ± 0.01^a^	4.21 ± 0.01^a^
Fortified oil stored at RT	62.11 ± 0.01^b^	1.71 ± 0.02^b^
Fortified oil stored at 37°C	60.01 ± 0.01^c^	1.60 ± 0.02^c^

*Note:* Data are expressed as mean ± SE. All analyses were done in triplicate. Mean values in the same column with different superscripts were significantly different at *p* < 0.05.

Abbreviation: RT, room temperature.

Similarly, at 6 months of storage, the vitamin D_3_ concentration in the oil had decreased significantly (*p* < 0.05) (Table 6). Also, at room temperature and 37°C storage, the vitamin D_3_ loss was 59% and 62%, respectively. Compared with vitamin A, apparently, the vitamin D_3_ loss upon the same storage period and temperature was higher. In fact, many researchers reported that both vitamin D_2_ and vitamin D_3_ are moderately stable in many foods during storage (Kolanowski and Berger 1999).

## 4 Conclusion

The study shed light on just how important food fortification is in the fight against micronutrient deficiencies, especially in low‐ and middle‐income countries like Ethiopia. Vitamins A and D, in particular, remain serious public health challenges. The research findings offer useful insights for making food fortification programs more effective, not just in Ethiopia but in similar contexts as well. One key takeaway is the need for local data to truly understand how well these efforts are working—especially when it comes to how much of the added vitamins are lost during cooking and storage. The results showed that vitamin A does not hold up well to high‐heat cooking, with a significant amount being lost. For best results, fortified oil should be used at temperatures below 200°C. Vitamin D_3_ also proved to be quite fragile, with large losses seen after 6 months of storage—highlighting the importance of using fortified oil within a shorter time frame. To make the most of these fortification efforts, more research is needed to adapt traditional Ethiopian cooking methods so that they preserve as many nutrients as possible.

## Ethics Statement

The authors have nothing to report.

## Conflicts of Interest

The authors declare no conflicts of interest.

## Data Availability

Data is available at Addis Ababa university as thesis (https://etd.aau.edu.et/bitstreams/a5eb7e75‐f8d0‐4142‐ae9e‐be6f3bd359dc/download).
